# A study of the DIBAL-promoted selective debenzylation of α-cyclodextrin protected with two different benzyl groups

**DOI:** 10.3762/bjoc.18.165

**Published:** 2022-11-17

**Authors:** Naser-Abdul Yousefi, Morten L Zimmermann, Mikael Bols

**Affiliations:** 1 Department of chemistry, University of Copenhagen, Universitetsparken 5, 2100, Copenhagen, Denmark

**Keywords:** aluminum hydrides, cyclodextrin, debenzylation, 2,4-dichlorobenzyl, selective

## Abstract

An α-cyclodextrin protected with 2,4-dichlorobenzyl groups on the primary alcohols and ordinary benzyl groups on the secondary alcohols was prepared and subjected to DIBAL (diisobutylaluminum hydride)-promoted selective debenzylation. Debenzylation proceeded by first removing two dichlorobenzyl groups from the 6^A,D^ positions and then removing one or two benzyl groups from the 3^A,D^ positions.

## Introduction

**Figure 1:**
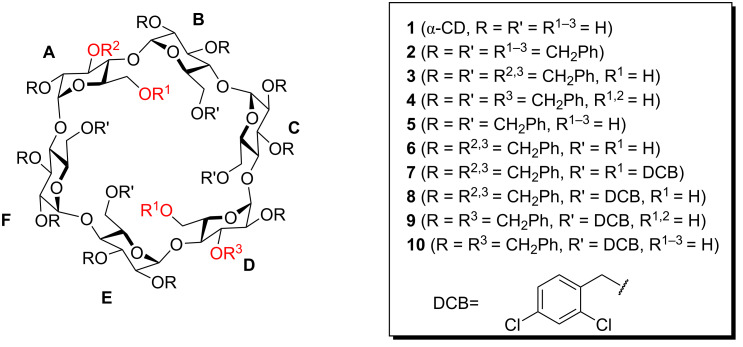
Structure of α-cyclodextrins **1**–**10**.

α-Cyclodextrin (**1**) is a cyclic carbohydrate consisting of six α-1,4-linked ᴅ-glucose molecules (Figure 1). It has a donut-like structure with the glucose residues all aligned with the α-side towards the center of the ring and the polar hydroxy groups pointing towards the sides [1]. This makes the ‘hole’ in the donut a lipophilic cavity that in water can form complexes with small hydrophobic molecules [2] driven by the entropy increase by expulsion of water [3]. Compound **1** has a wide range of applications where the complexation of substances such as pharmaceuticals or fragrances is exploited since it is cheap, harmless and biodegradable [4]. It is also a useful building block for sensors and/or capture devices, advanced materials, and even artificial enzymes.

Most such uses require that compound **1** can be chemically modified so that linkers, lids or catalytic groups can be installed which is no simple task due to the many similar functionalities in **1** [5–7]. A very useful way to access the hydroxy groups in a selective manner is the perbenzylation of **1** and the subsequent selective debenzylation of **2** using DIBAL [8–10]. This gives access to 6^A^-mono- and 6^A,D^ diol (**3**) in high yields and purity, and by extension of this method further deprotection on the primary [10–12] and secondary rim can be made [13–15]. The reaction of **2** with DIBAL leads quite rapidly to diol **3** and then much slower to triol **4** and tetrol **5**. These methods are so useful because virtually any chemical modification at the deprotected sites can be made followed by global deprotection of the *O*-benzyl groups with hydrogenolysis.

Recently, we observed a strong substituent effect when substituted benzyl groups were used in these reactions with electron-poor benzyl groups reacting much more sluggishly. Indeed the per-2,4-dichlorobenzyl (DCB) protected compound **1** was completely resistant to DIBAL even when treated for several days [15]. This led us to wonder if an α-cyclodextrin protected on the primary hydroxy groups with DCB groups and on the secondary hydroxy groups with ordinary benzyl groups would lead to selective debenzylation of one or more of the secondary hydroxy groups without the primary hydroxy groups being touched. In this work we have investigated this hypothesis and found that even when protected as DCB groups the primary alcohols are deprotected more readily than the secondary alcohols of **1**.

## Results and Discussion

**Scheme 1:**
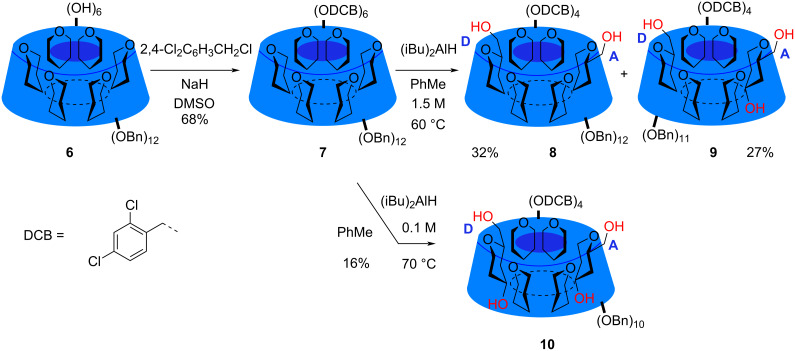
The reaction of perbenzylated α-cyclodextrin with iBu_2_AlH.

The starting point of the synthesis is the known partially benzylated derivative **6**, which according to the literature can be made either from **2** by selective acetolysis of all the primary benzyl groups and ester cleavage [16] or from **1** by selective protection of the primary OH groups with *tert*-butyldimethylsilyl groups, followed by benzylation and desilylation [17–18]. We used both methods to prepare **6**: The acetolysis method is convenient when perbenzyl α-cyclodextrin (**2**) is at hand but requires very strict temperature control during the acetolysis step. The silylation method requires careful drying of **1** before the silylation but is otherwise experimentally simple. Hexol **6** was then DCB-protected using 2,4-dichlorobenzyl chloride and sodium hydride in DMSO. As self-condensation of the alkylating agent is possible the reaction was carried out by mixing **6** and NaH in DMSO and then adding the 2,4-dichlorobenzyl chloride with a syringe pump over several hours. This gave the fully protected compound **7** in 68% yield (Scheme 1).

Reaction of **7** with DIBAL was carried out under a number of different conditions as listed in Table 1. Firstly, reaction with DIBAL in toluene at 0.3 M concentration and at 50 °C gave after 24 h almost complete conversion of the starting material to a symmetrical compound **8** that according to MALDI–TOF MS has lost two DCB groups and not any benzyl group.

**Table 1:** Reaction conditions for the partial debenzylation of **7**. The solvent was always toluene.

Entry	[Substrate]	[DIBAL]	*T* (°C)	Time (h)	Compounds^a^	Isolated (yield)

1	2.9 mM	0.3 M	50	24	**8** (93%) & monool 7%	–
2	14 mM	1.5 M	50	24	**8** (100%)	–
3	13 mM	1.5 M	60	24	**8** (51%) & **9** (49%)	**8** (32%) & **9** (27%)
4	1.3 mM	0.1 M	70	72	**8** (18%), **9** (55%) & **10** (27%)	–
5	1.3 mM	0.1 M	70	144	**8** (4%), **9** (32%) & **10** (64%)	**10** (16%)

^a^The ratio of compounds in the crude reaction product according to ^1^H NMR.

The compound was analyzed with ^1^H and ^13^C NMR (800/201 MHz), COSY, HSQC, TOCSY, and ROESY (Supporting Information File 1) which gave the NMR assignments shown in Table 2 and identification of **8** as the 6^A,D^ diol. The most significant observations in this assignment were 1) the compound is symmetric with only 3 different sugar residues which combined with the knowledge from MS that two DCB groups have been lost means that only the structure **8** is possible. 2) The residues with the lowest anomeric proton (δ 4.71) could be seen to correlate to the OH proton at δ 3.15 in TOCSY identifying them as **A**/**D**. 3) The residues with the highest anomeric proton (δ 5.70) could be seen to correlate to H-4 (at δ 3.80) of the **A**/**D** residues in ROESY identifying them as **B**/**E**. 4) The anomeric proton of the final residue (δ 4.73) correlated to H-4 at δ 3.92 in ROESY confirming them to be **C**/**F**.

**Table 2:** ^1^H and ^13^C NMR (800/201 MHz, CDCl_3_) chemical shifts of diol **8**.

	A/D^a^	B/E^a^	C/F^a^

H-1	4.71 (d)	5.70 (d, *J* = 4.0 Hz)	4.73 (d)
H-2	3.42 (dd)	3.58 (dd, *J* = 9.7, 3.9 Hz)	3.41 (dd)
H-3	4.10	4.21 (dd, *J* = 9.6, 7.6 Hz)	4.03
H-4	3.80 (t)	3.92	3.73
H-5	4.02	3.96	3.97
H-6a	3.73	4.08	3.94
H-6b	3.73	3.74	3.84
OH	3.15 (s)	–	–
Bn	5.43 (d, *J* = 10.3 Hz, 2H), 5.16 (d, *J* = 10.7 Hz, 2H), 4.88 (d, 2H), 4.87 (d, 2H), 4.82–4.68 (m, 6H), 4.60 (d, *J* = 13.3 Hz, 2H), 4.56–4.43 (m, 12H), 4.36 (2d, 4H)		
Ar		7.21 (m, 72H)	
C-1	98.4	98.2	98.3
C-2	80.1	77.8	79.1
C-3	81.6	80.9	80.7
C-4	75.0	81.3	81.9
C-5	71.5	71.8	72.2
C-6	62.4	70.3	70.9
Bn	76.6, 76.2, 74.1, 73.6, 73.2, 72.6, 70.00, 69.95		
Ar	139.31, 139.27, 139.26, 138.7, 138.3, 138.0 (ipsoC Ph), 134.5, 134.3, 134.1, 133.8, 133.6, 133.3 (ipsoC DCB), 130.0, 129.9, 129.2, 129.1, 128.5, 128.5, 128.3, 128.24, 128.21, 128.18, 128.18, 128.16, 128.0, 127.9, 127.8, 127.37, 127.35, 127.3, 127.24, 127.21, 127.17, 127.0, 126.5 (CH Ph & DCB)		

^a^The letters A to F refer to each of the monosaccharides using normal cyclodextrin nomenclature as of Figure 1.

Remaining in the reaction mixture was some of the monool though this compound was not isolated and identified. Carrying out the same reaction with 1.5 M DIBAL gave complete conversion to **8** (Table 1, entry 2). The formation of **8** from **7** is surprising because it contrasts the complete lack of reaction of fully DCB protected **1** with DIBAL [15]. It means that the identity of protective groups on the secondary rim influence the reaction at the primary rim significantly, most probably by a collective inductive effect.

When the reaction of **7** with DIBAL was carried out at higher temperature further debenzylation was observed with, according to MS, a triol **9** being formed from **8**. When **7** was reacted with 1.5 M DIBAL in toluene at 60 °C for 24 hours an almost equal amount of **8** and **9** was present (Table 1, entry 3) and 27% of triol **9** together with 32% of **8** was isolated. A tetrol **10** was obtained upon even longer reaction of **7**: If reacted with 0.1 M DIBAL in toluene for 3 days at 70 °C a mixture of 18% of **8**, 55% of **9**, and 27% of **10** was seen (Table 1, entry 4). When the time was extended to 6 days **10** was the predominant compound (Table 1, entry 5) and could be isolated in 16% yield. Triol **9** was identified using ^1^H and ^13^C NMR (800/201 MHz), COSY, HSQC, HMBC, TOCSY, and ROESY (Supporting Information File 1) leading to NMR assignments shown in Table 3 and identification as the 3^A^,6^A,D^ triol. The most significant observations in this assignment were 1) MS showed the compound had lost a benzyl group from the structure of **8**. 2) One of the residues (A) which have an anomeric proton at δ 4.80 is seen on TOCSY and COSY to correlate to a H-3 signal at δ 4.22 which has a corresponding carbon at δ 73.5. This carbon is 7–8 ppm lower than other C-3 signals revealing that it is not alkylated. 3) This same residue (A) has an outlying H-2 proton signal at δ 3.28 which correlates in TOCSY to an OH proton at δ 2.80. 4) A HMBC correlation connects the carbon signal at δ 62.3 to the unusual H-4 signal at δ 3.35 in residue A. This with the knowledge that **9** is formed from **8** gives the structure of **9**. Overall the spectrum of **9** resembles that of the benzylated triol [13] **4** and the assignment is not surprisingly very similar [15].

**Table 3:** ^1^H and ^13^C NMR (800/201 MHz, CDCl_3_) chemical shifts of triol **9**.

	A^a^	B^a^	C^a^	D^a^	E^a^	F^a^

H-1	4.80	5.08	4.71	4.76	5.58	4.78
H-2	3.28	3.62	3.38	3.45	3.56	3.39
H-3	4.22	4.26	4.01	4.12	4.11	4.03
H-4	3.35	3.82	3.76	3.74	3.88	3.63
H-5	3.86	3.88	3.97^b^	4.15^b^	3.97	3.97^b^
H-6a	3.70	4.06	3.94^c^	3.73	4.12^c^	3.88^c^
H-6b	3.63	3.72	3.82^c^	3.73	3.71^c^	3.77^c^
OH	2.80 (bs, 1H)	–	–	2.98 (bs, 1H)	–	–
Bn	5.51 (d, *J* = 10.8 Hz, 1H), 5.28 (d, *J* = 10.6 Hz, 1H), 5.17 (d, *J* = 10.5 Hz, 1H), 5.13 (d, *J* = 11.2 Hz, 1H), 4.96 (d, *J* = 11.0 Hz, 1H), 4.90 (2d, 2H), 4.84 (d, *J* = 11.5 Hz, 1H), 4.82–4.68 (m, 4H), 4.62–4.38 (m, 18H)					
Ar	7.42 (m,2H), 7.35 (m, 4H), 7.32–7.08 (m, 61H)					
C-1	100.4	100.8	99.6	98.0	98.7	98.4
C-2	77.5	78.2	78.7	80.0	77.6	79.6
C-3	73.5	81.2	80.8	80.6^d^	81.5^d^	80.6
C-4	82.1	82.4	76.5^e^	81.5^e^	80.9	82.7
C-5	71.8	72.2	71.8^f^	71.5	71.8^f^	72.4^f^
C-6	62.3	70.2	71.1^g^	62.5	70.0^g^	71.2^g^
Bn	76.62, 76.47, 76.03, 76.01, 74.52, 74.31, 73.48, 73.01, 72.96, 72.78, 72.62, 71.99, 69.96, 69.88, 69.82					
Ar	139.7–137.6 (Ph ipso), 134.5–133.3 (Ar ipso), 129.9–126.6 (Ph & Ar CH)					

^a^The letters A to F refers to each of the monosaccharides using normal cyclodextrin nomenclature as of Figure 1. ^b^Shifts may be interchanged. ^c^Shifts may be interchanged. ^d^Shifts may be interchanged. ^e^Shifts may be interchanged. ^f^Shifts may be interchanged. ^g^Shifts may be interchanged.

Similar NMR analysis of **10** gave the assignments shown in Table 4. The most significant observations in this structural assignment were 1) MS showed the compound had lost two benzyl groups from the structure of **8** and from NMR it was found to be symmetrical. 2) The residues (A/D) which have an anomeric proton at δ 4.79 is seen on TOCSY and COSY to correlate a H-3 signal at δ 4.28 which has a corresponding carbon signal at δ 73.0 revealing that C-3 is debenzylated. This with the symmetry of the compound and the knowledge it is formed from **9** gives the structure. 3) HMBC correlations between C-1 and H-4 in the former glucose (101.8 → 3.40, 101.2 → 3.65, 100.2 → 3.81) gave the order of residues. Overall the spectrum of **10** resembles that of the fully benzylated tetrol **5** [13].

**Table 4:** ^1^H and ^13^C NMR (800/126 MHz, CDCl_3_) chemical shifts of tetrol **10**.

	A/D^a^	B/E^a^	C/F^a^

H-1	4.79	4.92	4.70
H-2	3.29	3.60	3.33
H-3	4.28	4.17	3.96
H-4	3.40	3.81	3.65
H-5	3.89	3.88	3.88
H-6a	3.67	3.80	4.04
H-6b	3.67	3.79	3.67
Bn	5.42 (d, *J* = 11.0 Hz, 2H), 5.21 (d, *J* = 11.2 Hz, 2H), 4.95–4.16 (m, 24H)		
Ar	7.53–6.93 (m, 62H)		
C-1	101.2	101.8	100.2
C-2	77.5	78.8	79.1
C-3	73.0	81.4	80.8
C-4	83.7	82.4	82.7
C-5	72.0^b^	72.3^b^	71.4^b^
C-6	62.1	70.9	70.0
Bn	76.5, 76.0, 74.7, 72.7, 72.6, 69.9, 69.8		
Ar	139.6, 139.5, 138.6, 138.5, 137.3 (Ph ipso), 134.5, 134.4, 134.0, 133.8, 133.4, 133.2 (Ar ipso), 129.8, 129.8, 129.1, 129.05, 128.9, 128.73, 128.69, 128.5, 128.4, 128.36, 128.32, 128.28, 128.23, 128.15, 128.1, 127.8, 127.7, 127.5, 127.3, 127.24, 127.15, 127.1 (Ar & Ph CH)		

^a^The letters A to F refers to each of the monosaccharides using normal cyclodextrin nomenclature as of Figure 1. ^b^Shifts may be interchanged.

As **9** and **10** are analogues to the products formed from **2** this means that **7** is debenzylated almost similarly other than the DCB removal at the primary rim is somewhat slower. Debenzylation on the secondary rim does not occur before DCB groups have been removed on the primary side supporting the hypothesis that the debenzylation on the secondary side is intramolecular or at least directed by alkoxy aluminate at O6.

## Conclusion

It is clear that the O2,O3-DCB groups in fully DCB-protected **1** are affecting the DIBAL-promoted debenzylation tremendously: When present no reaction is observed and when exchanged with unsubstituted benzyl groups debenzylation occurs following the already known pattern. The reason for this behavior is probably due to the collective electron-withdrawing effect of the chlorine atoms making the glucose residues of the fully DCB-protected compound more electron deficient at the ring oxygen. This means that the Lewis acidic aluminum reagent has more difficulty binding to this oxygen which is important in the mechanism [9,15].

## Experimental

**General information:** In a manner similar to [15] dry solvents were tapped from a PureSolv solvent purification system. Reactants were purchased from commercial sources and used without further purification. HRMS were recorded on a Bruker Solarix XR mass spectrometer analyzing TOF. Generally, NMR spectra were recorded on a 500 MHz Bruker instrument with a cryoprobe. The 800 MHz spectra were recorded at 25 °C on a Bruker Avance Neo spectrometer with 5 mm CPTXO Cryoprobe C/N-H-D optimized for direct ^13^C detection. Chemical shifts (δ) are reported in ppm relative to the residue solvent signals or other solvent present. Flash chromatography was carried out on a Büchi Pure Chromatography Instrument C-805 using silica gel columns.

**6^A–F^-Hexa-*O*-(2,4-dichlorobenzyl)-2^A–F^,3^A–F^-dodeca-*O*-benzyl-α-cyclodextrin (7):** NaH (60% dispersion in mineral oil, 162 mg, 4.05 mmol) was added to a solution of hexol **6** (694 mg, 0.338 mmol) in anhydrous DMSO (20 mL) under a nitrogen atmosphere at room temperature. After bubbling had subsided, 2,4-dichlorobenzyl chloride (0.563 mL, 4.05 mmol) was added over four hours with a syringe pump. The mixture was left to stir overnight, and the reaction was quenched by addition of MeOH (10 mL). The mixture was diluted with toluene, and the organic phase washed with H_2_SO_4_ (1 M, 20 mL), then brine (3 × 20 mL), dried (MgSO_4_), filtered, and concentrated. Column chromatography in a solvent gradient of heptane/EtOAc 1:0 to 0:1 gave product **7** (693 mg, 0.230 mmol, 68%). ^1^H NMR (CDCl_3_, 500 MHz) δ 7.21–7.07 (m, 78H, Ar), 5.17 (d, *J* = 10.9 Hz, 1H, ArCH, 6H), 5.08 (d, *J*_1,2_ = 3.4 Hz, 1H, 6H, H-1) , 4.86 (d, *J* = 10.9 Hz, 6H, ArCH), 4.52–4.41 (m, 18H, 3 × ArCH), 4.37 (d, *J* = 13.4 Hz, 6H, ArCH), 4.16–4.08 (m, 12H, H-3, H-6a), 4.02–3.94 (m, 12H, H-4, H-5), 3.59 (d, *J* = 10.6 Hz, 6H, H-6b), 3.47 (dd, *J*_2,3_ = 9.8 Hz, 6H, H-2) ppm; ^13^C NMR (126 MHz, CDCl_3_) δ 139.17, 138.23, 134.35, 133.79, 132.95, 129.46, 128.98, 128.20, 128.06, 127.73, 127.51, 127.29, 127.07 (Ar), 98.75 (C-1), 80.90 (C-3), 79.37 (C-4), 78.97 (C-2), 75.66 (ArCH_2_), 72.93 (ArCH_2_), 71.62 (C-5), 69.94 (ArCH_2_) 69.91 (C-6) ppm; HRMS–MALDI^+^ (*m*/*z*): [M + Na]^+^ calcd for C_162_H_156_Cl_12_O_30_Na^+^, 3030.6782; found, 3030.67364.

**General procedure for the reactions of 7 with DIBAL:** A sample of compound **7** (65 mg, 22 μmol) was dissolved in 0 to 6 mL anhydrous toluene in a dry round-bottomed flask fitted with a septum and a stirring bar under nitrogen. Then, 1.5 mL DIBAL as a 1.5 M solution in toluene were added with a syringe. The reaction mixture was stirred at fixed temperature (50–70 °C) controlled by an oil bath. At the end of the reaction, the flasks’ contents were diluted with 20 mL toluene and was washed with 20 mL 1 M H_2_SO_4_ and water in a separating funnel. The organic layer was dried with sodium sulfate, filtered, concentrated, and analyzed by ^1^H NMR in CDCl_3_. The relative content of **7**, monool, **8**, **9**, and **10** in the sample was determined by comparing the integrals of peaks at δ 5.70 (d, 2H, H-1^BE^, **8**), 5.58 (d, 1H, H-1^E^, **9**), 5.50 (d, 1H, monool), 5.42 (d, 2H, Bn, **10**) and 5.08 (d, 6H, **7**) ppm.

**6^B,C,E,F^-Tetra-*O*-(2,4-dichlorobenzyl)-2^A–F^,3^A–F^-dodeca-*O*-benzyl-α-cyclodextrin (8) and 6^B,C,E,F^-tetra-*O*-(2,4-dichlorobenzyl)-2^A–F^,3^B–F^-undeca-*O*-benzyl-α-cyclodextrin (9):** Compound **7** (200 mg, 66.5 μmol) was dissolved in DIBAL-H (1.5 M in toluene, 5 mL, 7.5 mmol) in a flask under nitrogen and stirred at 60 °C for 24 hours. Methanol (1 mL) was slowly added and the solution was poured into 20 mL H_2_SO_4_ (1 M) and toluene (20 mL). The layers were partitioned, and the organic layer washed with saturated aqueous NaHCO_3_ (20 mL), dried with Na_2_SO_4_, filtered, and concentrated. Column chromatography in a solvent gradient of heptane/EtOAc 1:0 to 0:1 gave consecutively **8** (56 mg, 21 μmol, 32 %) and **9** (47 mg, 18 μmol, 27%). NMR data and assignments see Table 2 (**8**) and Table 3 (**9**). HRMS–MALDI (*m*/*z*): for **8** [M + Na]^+^ calcd for C_141_H_142_Cl_8_O_30_Na^+^, 2712.7431; found: 2712.74127. for **9** [M + K]^+^ calcd for C_162_H_156_Cl_12_O_30_K^+^, 2638.6701; found, 2638.71363.

**6^B,C,E,F^-Tetra-*O*-(2,4-dichlorobenzyl)-2^A–F^,3^B,C,E,F^-dodeca-*O*-benzyl-α-cyclodextrin (10):** Compound **7** (59 mg, 20 μmol) was dissolved in anhydrous toluene (14 mL) and DIBAL (1.5 M in toluene, 1 mL, 1.5 mmol) was added. The reaction mixture was stirred at 70 °C under nitrogen atmosphere. After 6 days the reaction mixture was diluted with toluene (25 mL) and quenched by addition of H_2_SO_4_ (1 M, 25 mL), then the organic layer was washed with water (25 mL), dried (Na_2_SO_4_), and concentrated to a crude product (35 mg) that according to NMR contained 4% **8**, 32% **9**, and 64% **10**. Flash chromatography in a EtOAc/heptane going from 1:3 to 1:1 gave **10** (8 mg, 3 μmol, 16%). NMR data and assignment see Table 4. HRMS–MALDI^+^ (*m*/*z*): [M + H]^+^ calcd for C_134_H_137_Cl_8_O_30_^+^, 2511.6706 (71.9%); found, 2511.69097.

## Supporting Information

File 1 Copies of NMR spectra of compounds **7**–**10**.
