# Supplementary material for: A study of the DIBAL-promoted selective debenzylation of α-cyclodextrin protected with two different benzyl groups
Source: Beilstein J Org Chem. 2022 Nov 17;18:1553–9. doi: 10.3762/bjoc.18.165 (PMC9679596; doi:10.3762/bjoc.18.165)

BEILSTEIN JOURNAL OF ORGANIC CHEMISTRY

Supporting Information

for

A study of the DIBAL-promoted selective debenzylation of α-cyclodextrin protected with two different benzyl groups

Naser-Abdul Yousefi, Morten L. Zimmermann and Mikael Bols

Beilstein J. Org. Chem. **2022,** *18,* 1553–1559. doi:10.3762/bjoc.18.165

Copies of NMR spectra of compounds 7–10

License and Terms: This is a supporting information file under the terms of the Creative Commons Attribution License (https://creativecommons.org/licenses/by/4.0). Please note that the reuse, redistribution and reproduction in particular requires that the author(s) and source are credited and that individual graphics may be subject to special legal provisions.
The license is subject to the *Beilstein Journal of Organic Chemistry* terms and conditions: (https://www.beilstein-journals.org/bjoc/terms)

Table of contents

1. Copies of NMR spectra of **7** S2
2. Copies of NMR spectra of **8** S3
3. Copies of NMR spectra of **9** S6
4. Copies of NMR spectra of **10** S9

^{1}H NMR (500 MHz, $CDCl_3$) of **7**

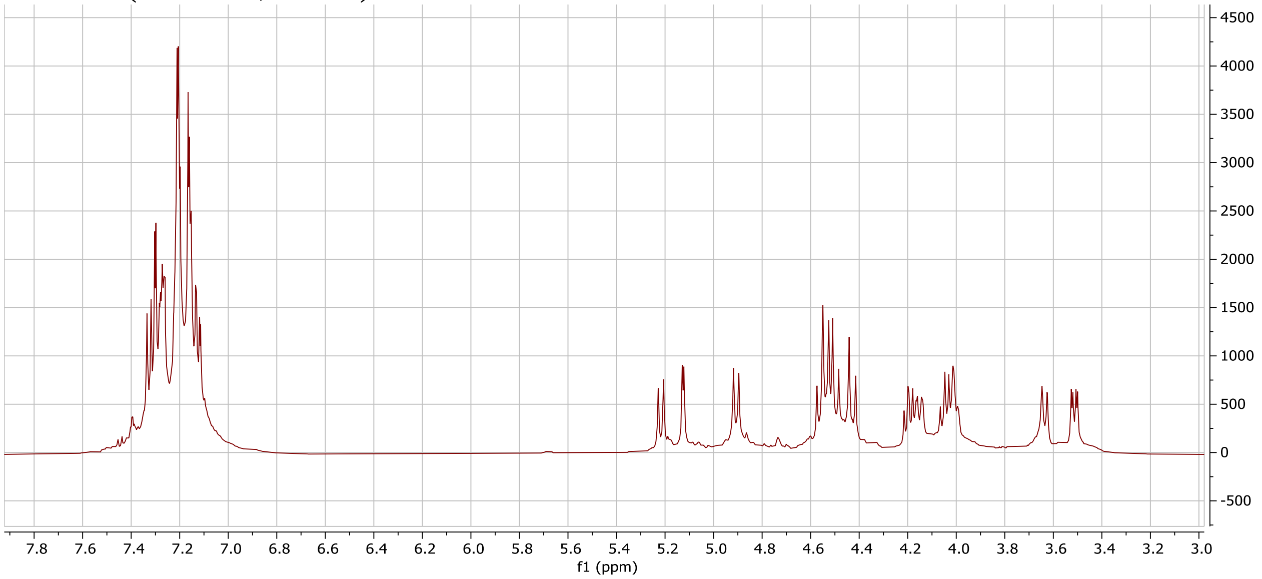

^{13}C NMR (126 MHz, $CDCl_3$) of **7**

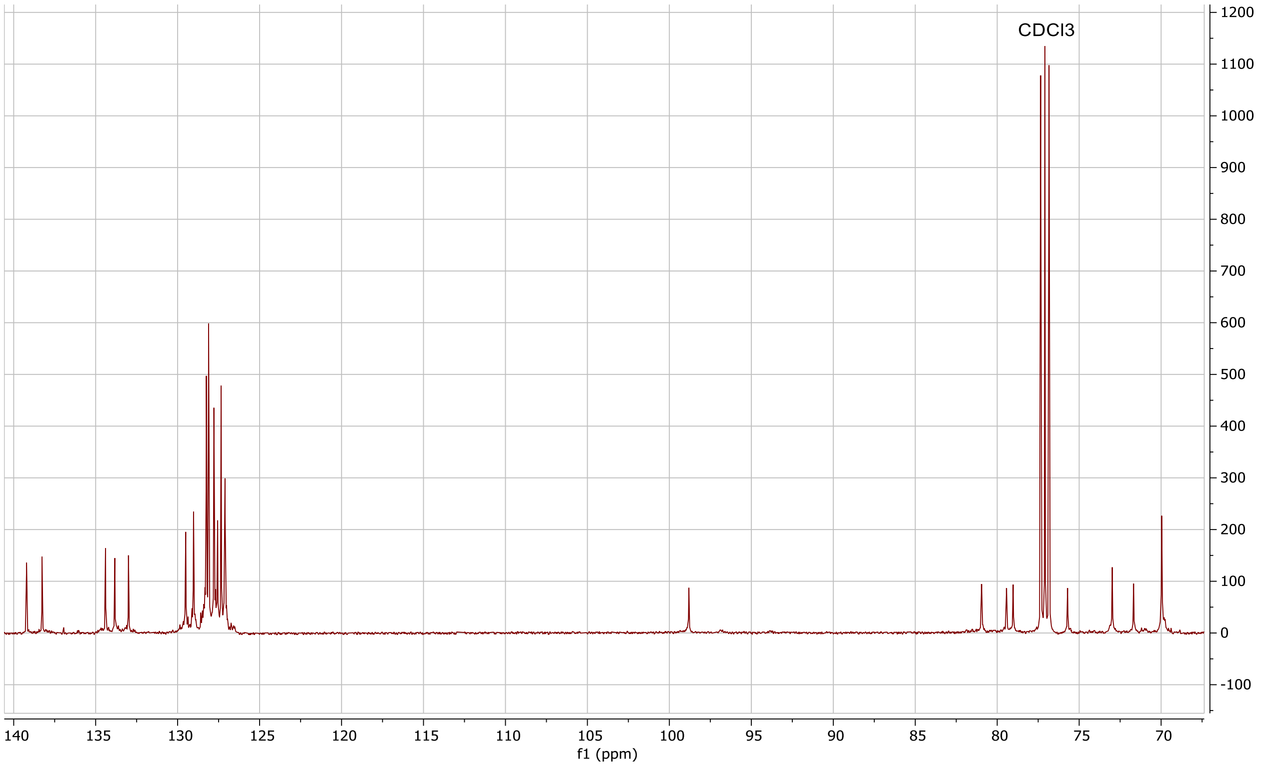

^{1}H NMR (800 MHz, $CDCl_3$) of **8**

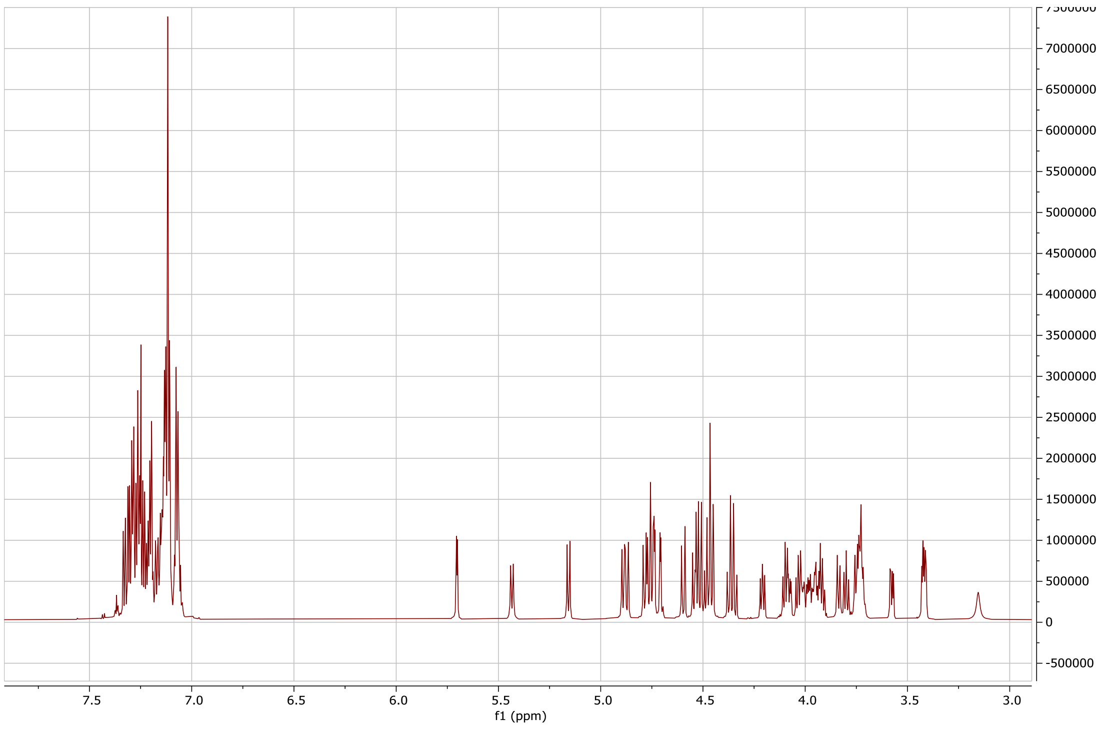

^{13}C NMR (200 MHz, $CDCl_3$) of **8**

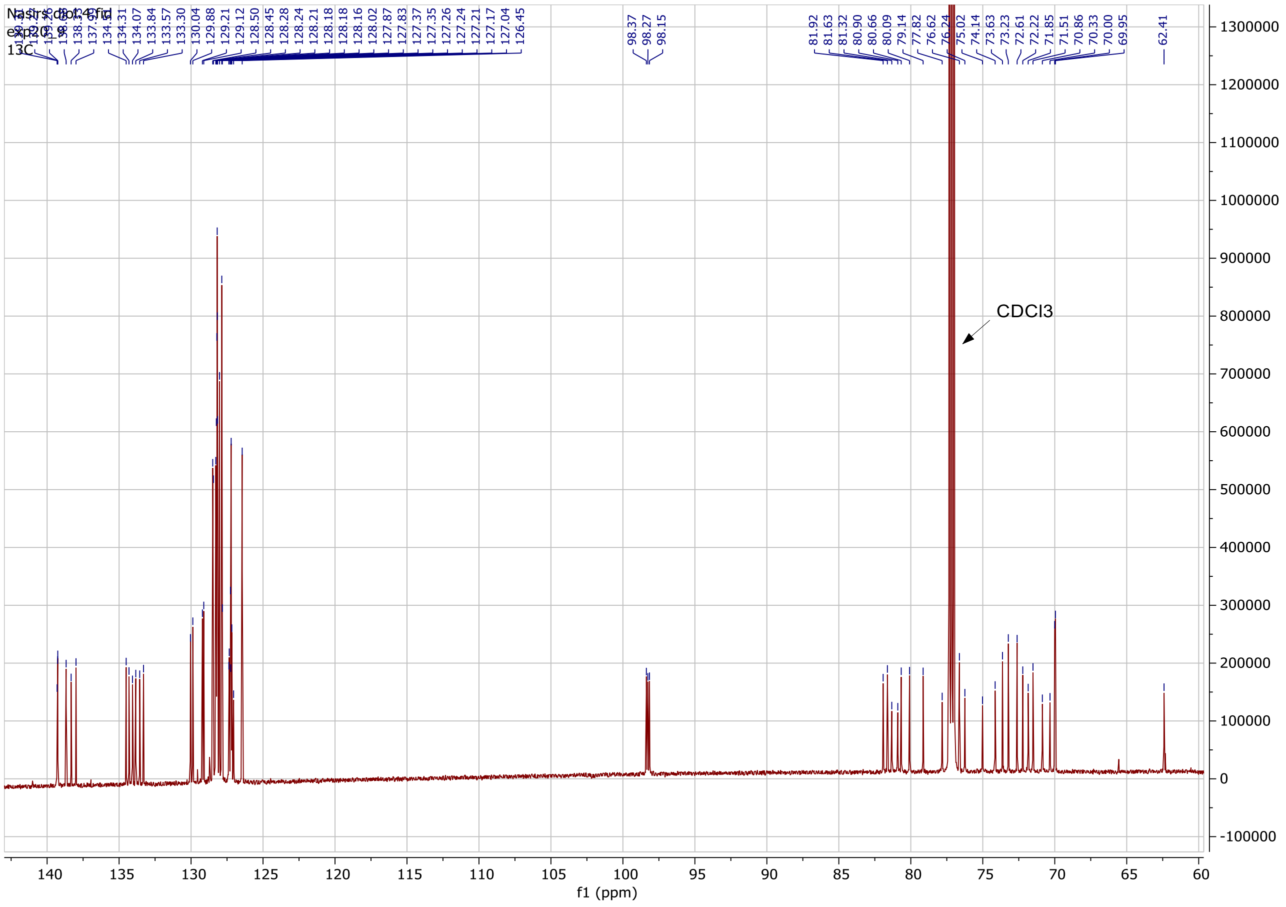

COSY (800 MHz, $CDCl_3$) of **8** (δ 2.5–6.0; aromatic signals not included)

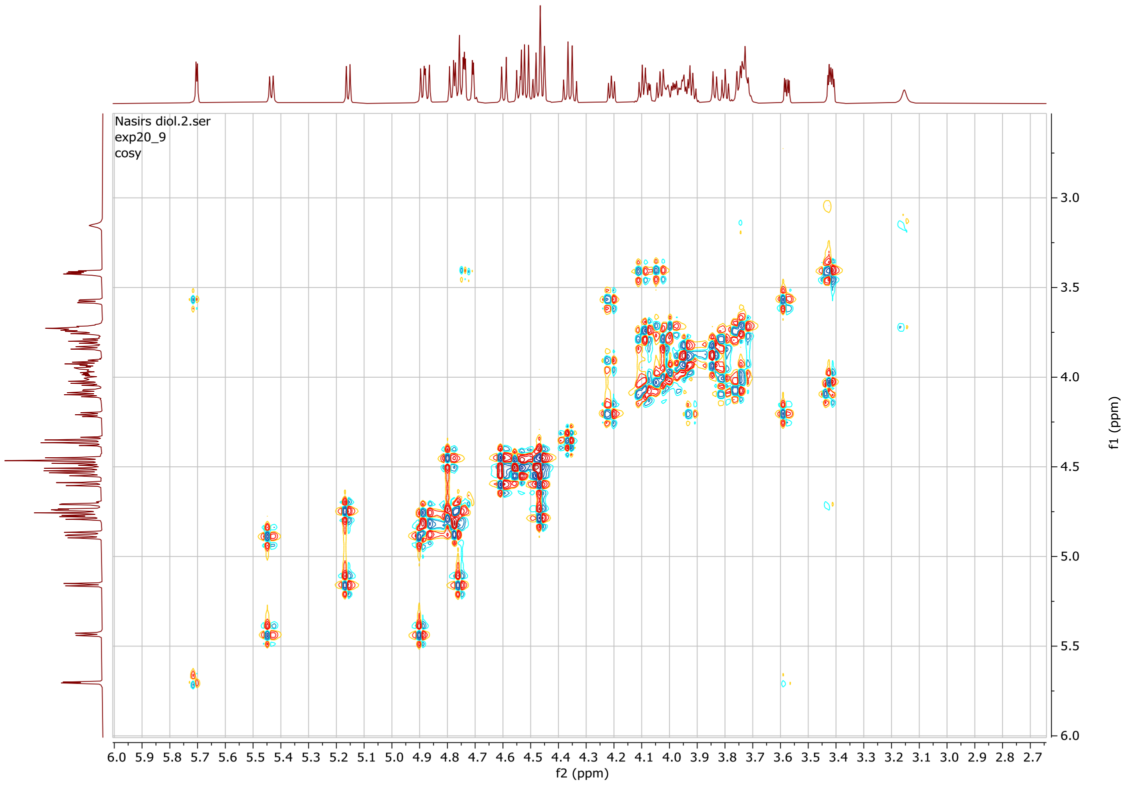

HSQC ($CDCl_3$) of **8** (aromatic signals not shown, anomeric signal folded in, offset −80 ppm)

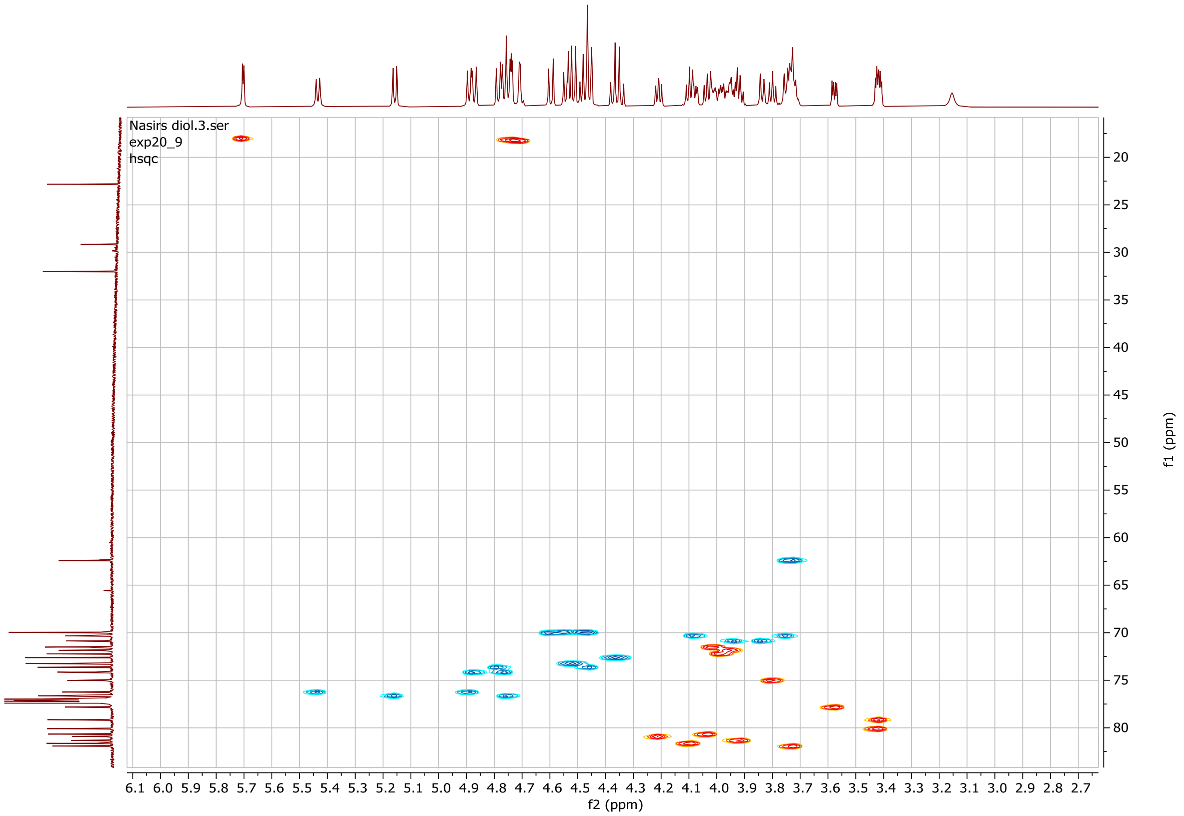

TOCSY (800 MHz, $CDCl_3$) of **8** (δ 2.5–6.0; aromatic signals not shown)

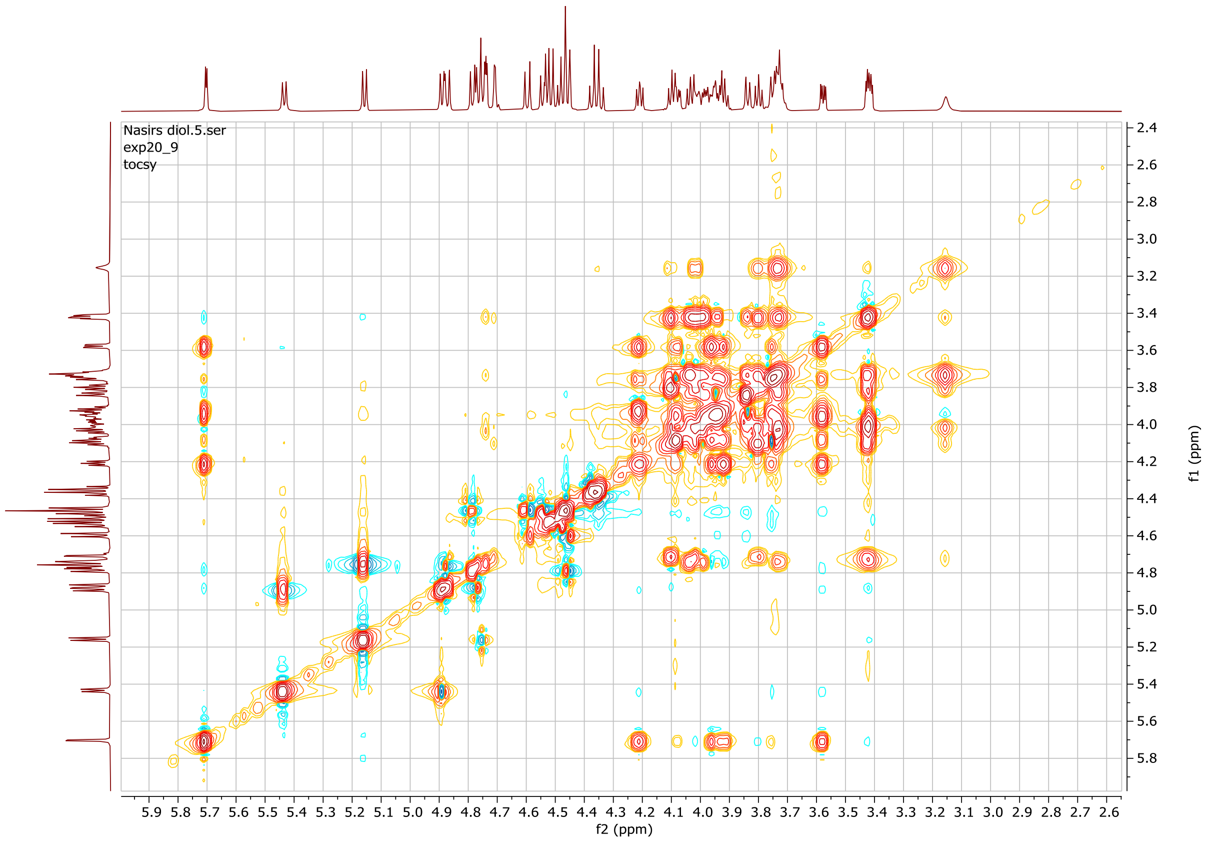

ROESY ($CDCl_3$) of **8** (aromatic signals not shown)

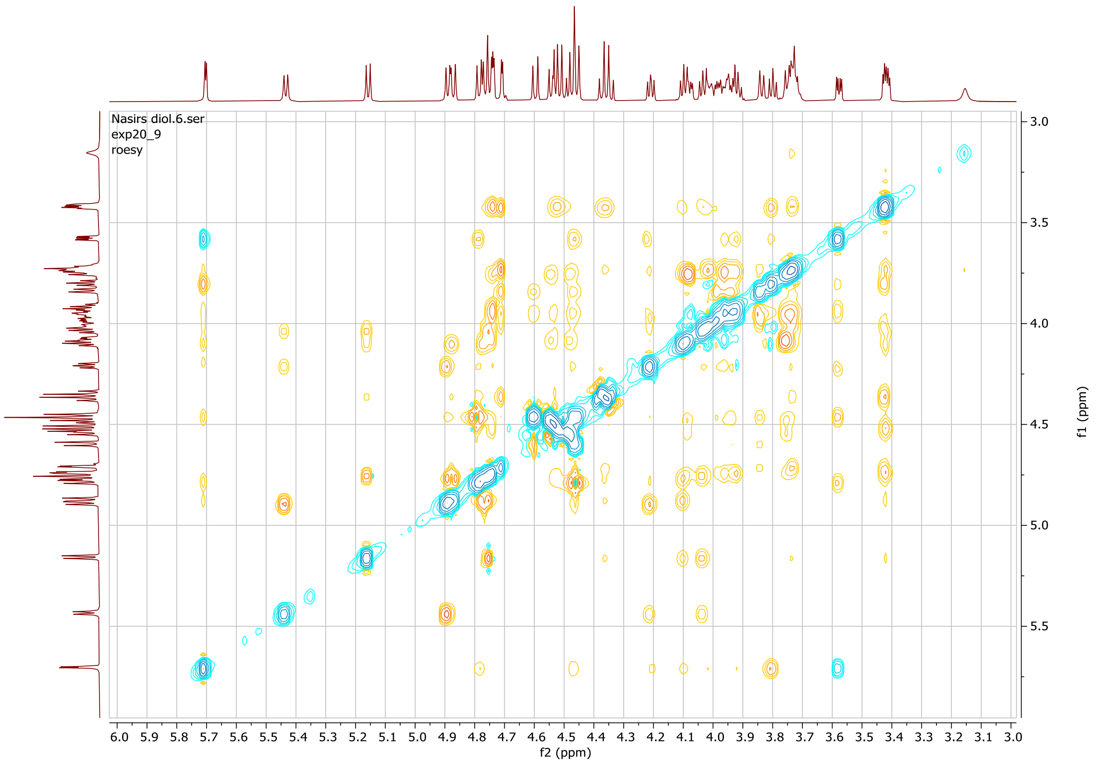

^{1}H NMR (800 MHz, $CDCl_3$) of **9**

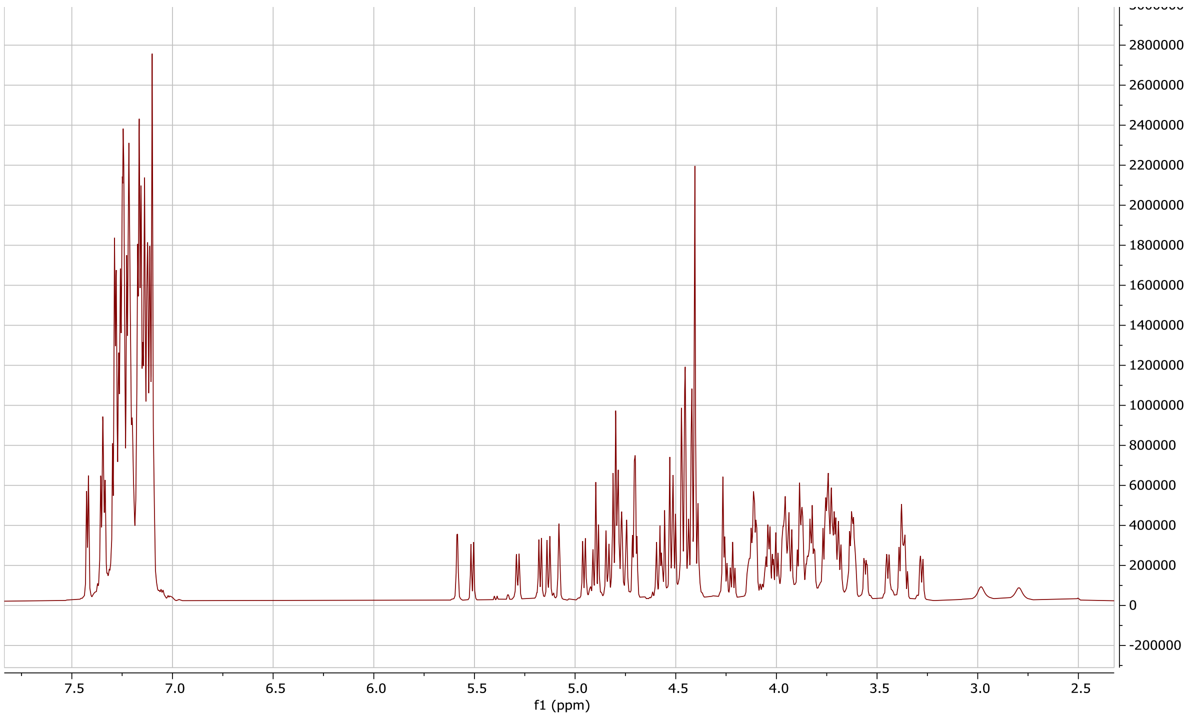

^{13}C NMR (200 MHz, $CDCl_3$) of **9**

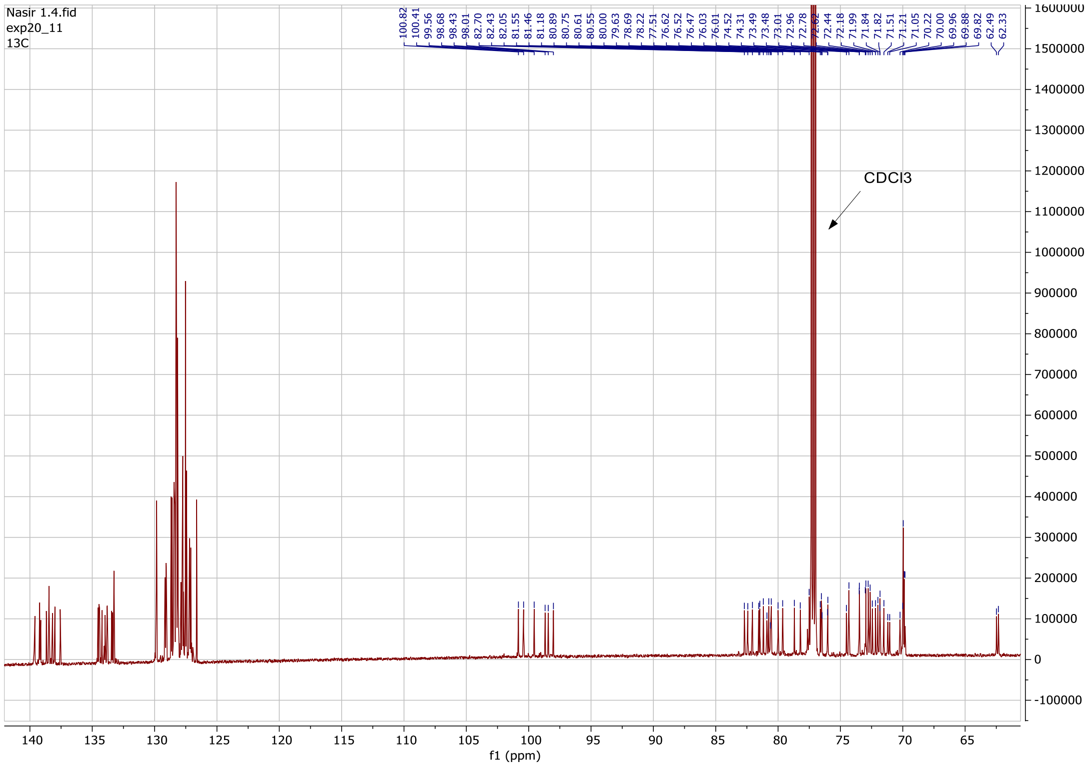

COSY (800 MHz, $CDCl_3$) of **9** (δ 2.5–6.0; aromatic signals not shown)

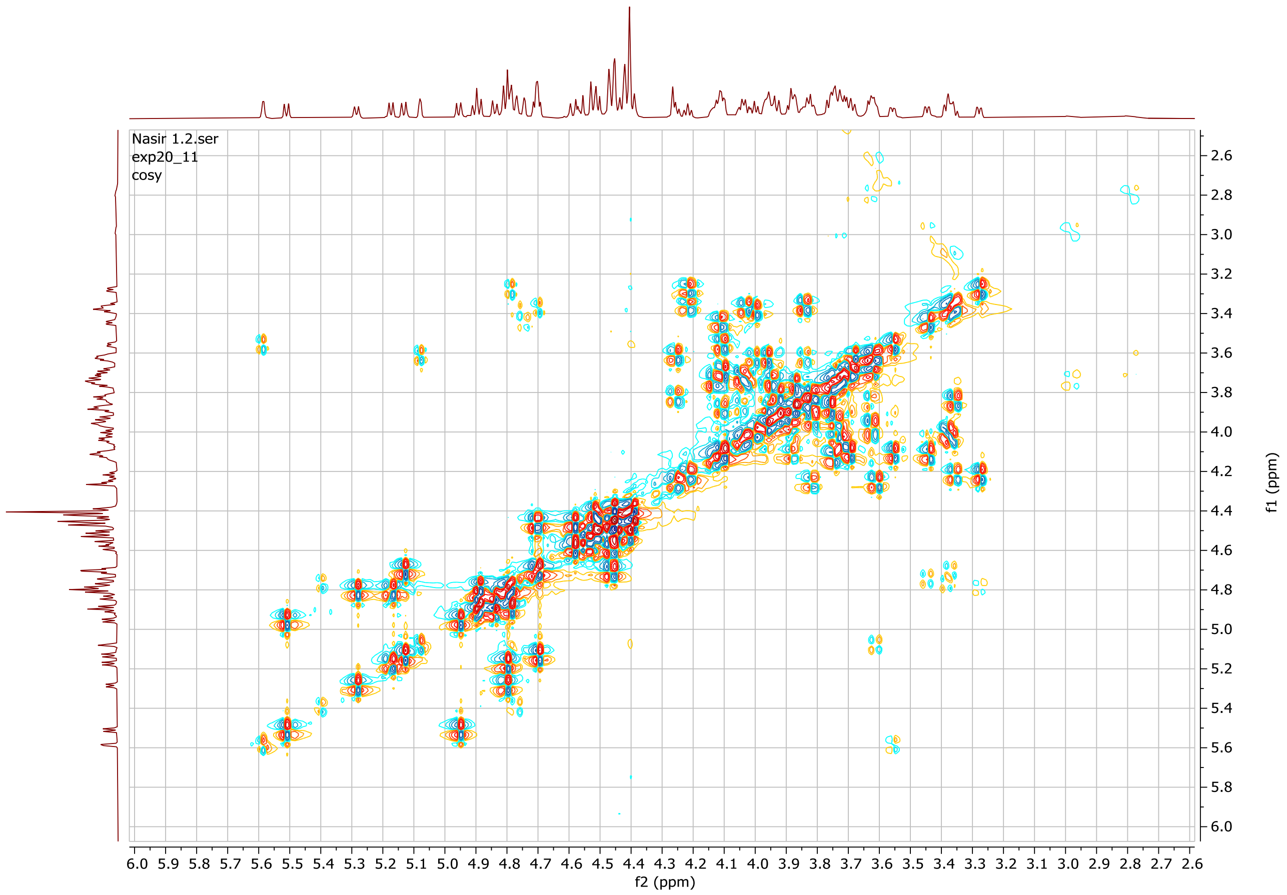

HSQC ($CDCl_3$) of **9** (aromatic signals not shown, anomeric signal folded in, offset −80 ppm)

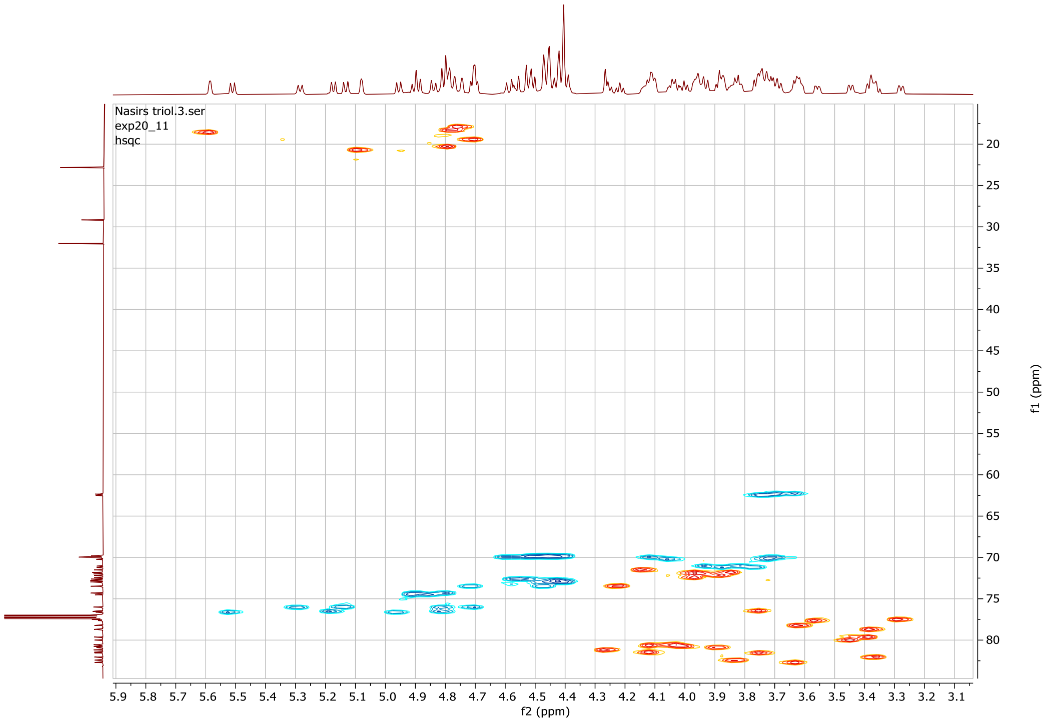

TOCSY (800 MHz, $CDCl_3$) of **9** (δ 2.5–6.0; aromatic signals not shown)

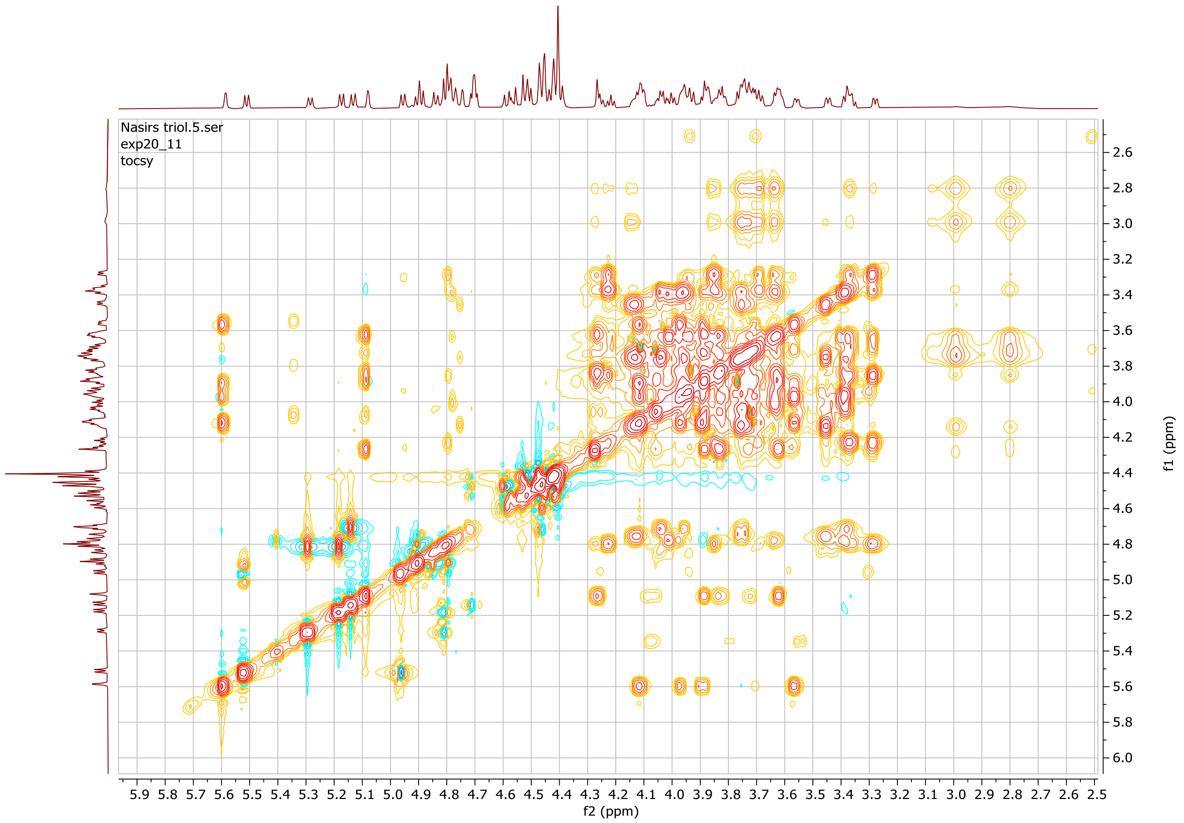

ROESY (800 MHz, $CDCl_3$) of **9** ((δ 2.5–6.0; aromatic signals not shown)

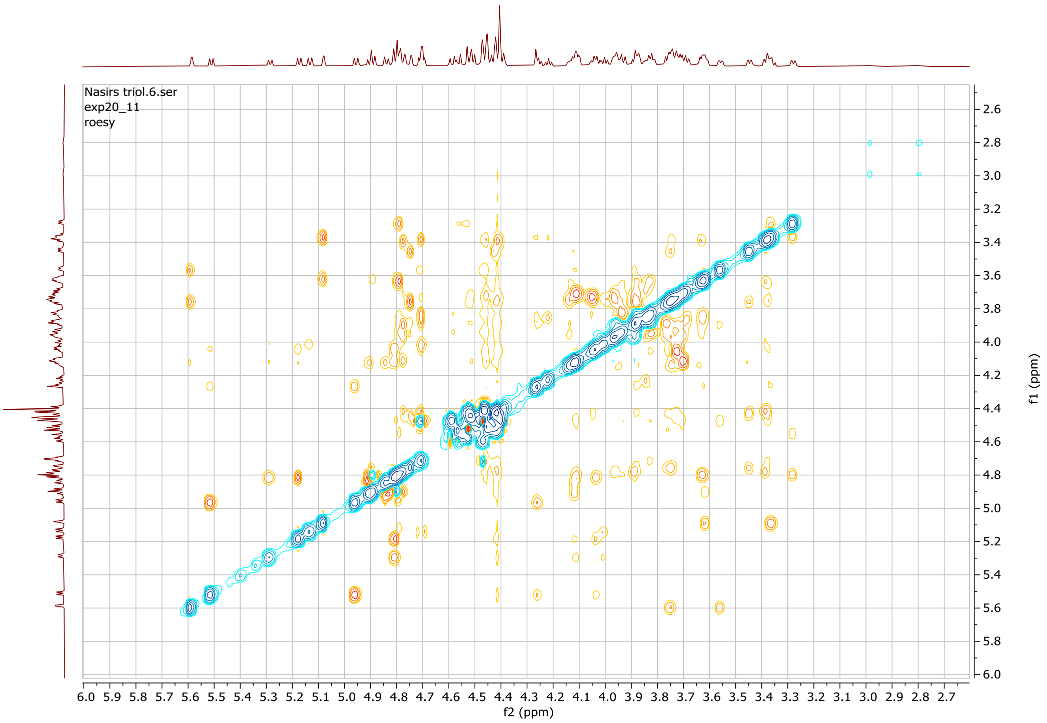

^{1}H NMR (800 MHz, $CDCl_3$) of **10** (δ 2.9–7.6)

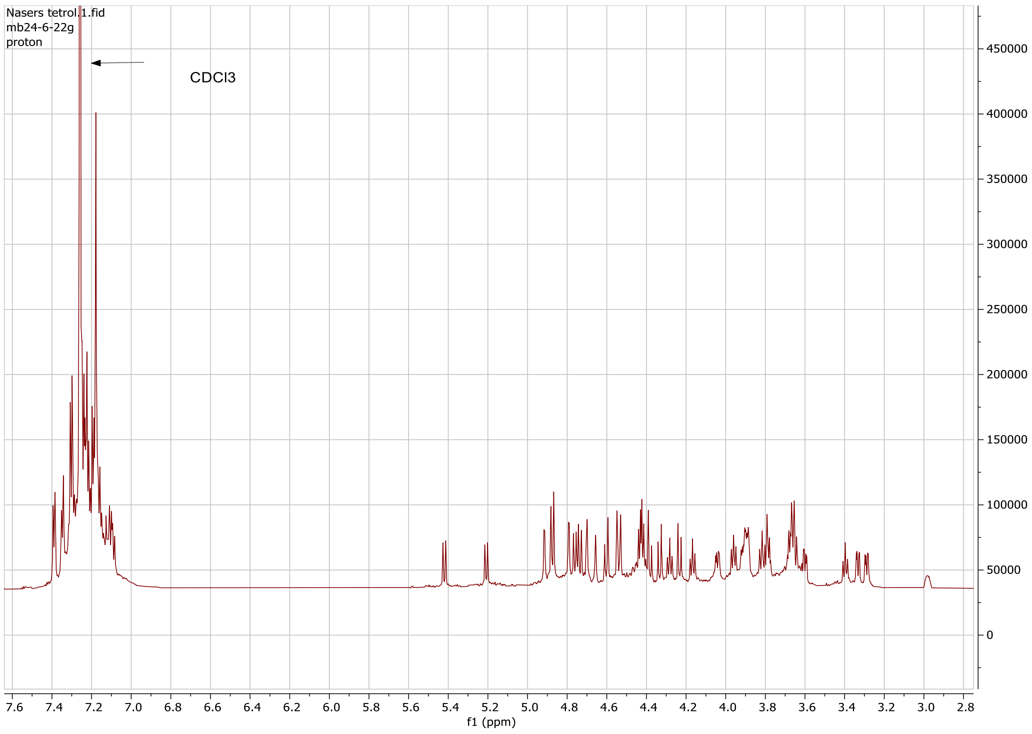

COSY (800 MHz, $CDCl_3$) of **10** (δ 2.5–6; aromatic signals not shown)

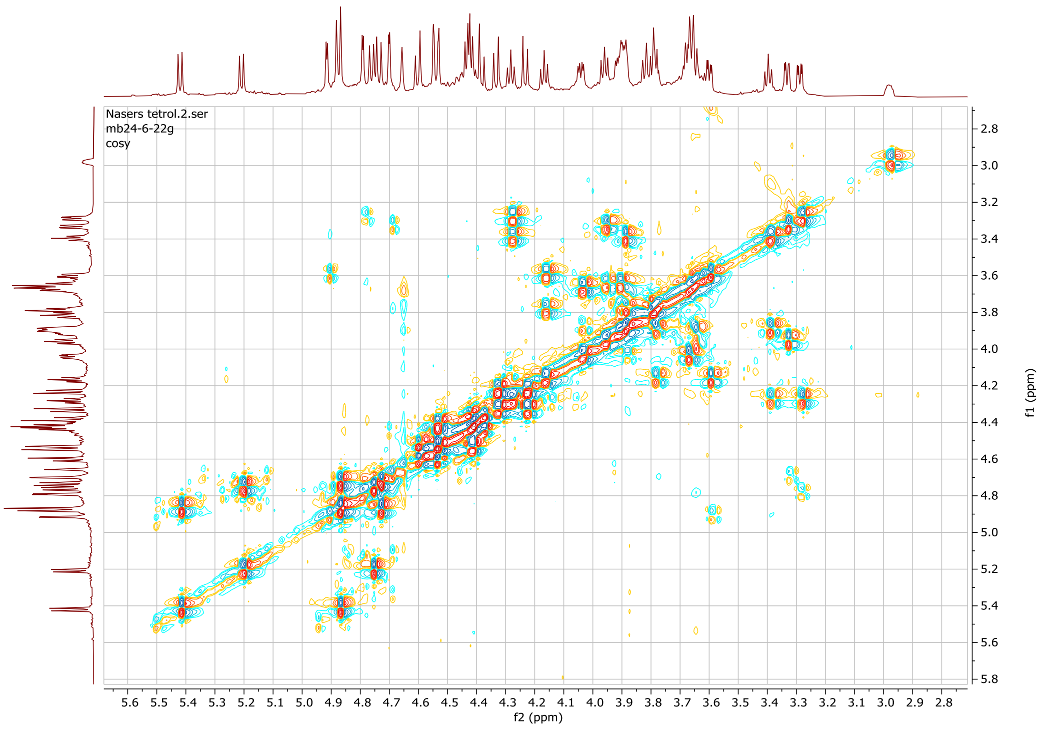

HSQC (800 MHz, $CDCl_3$) of **10** (δ 3–5.5 vs 60–110 ppm; aromatic signals not shown)

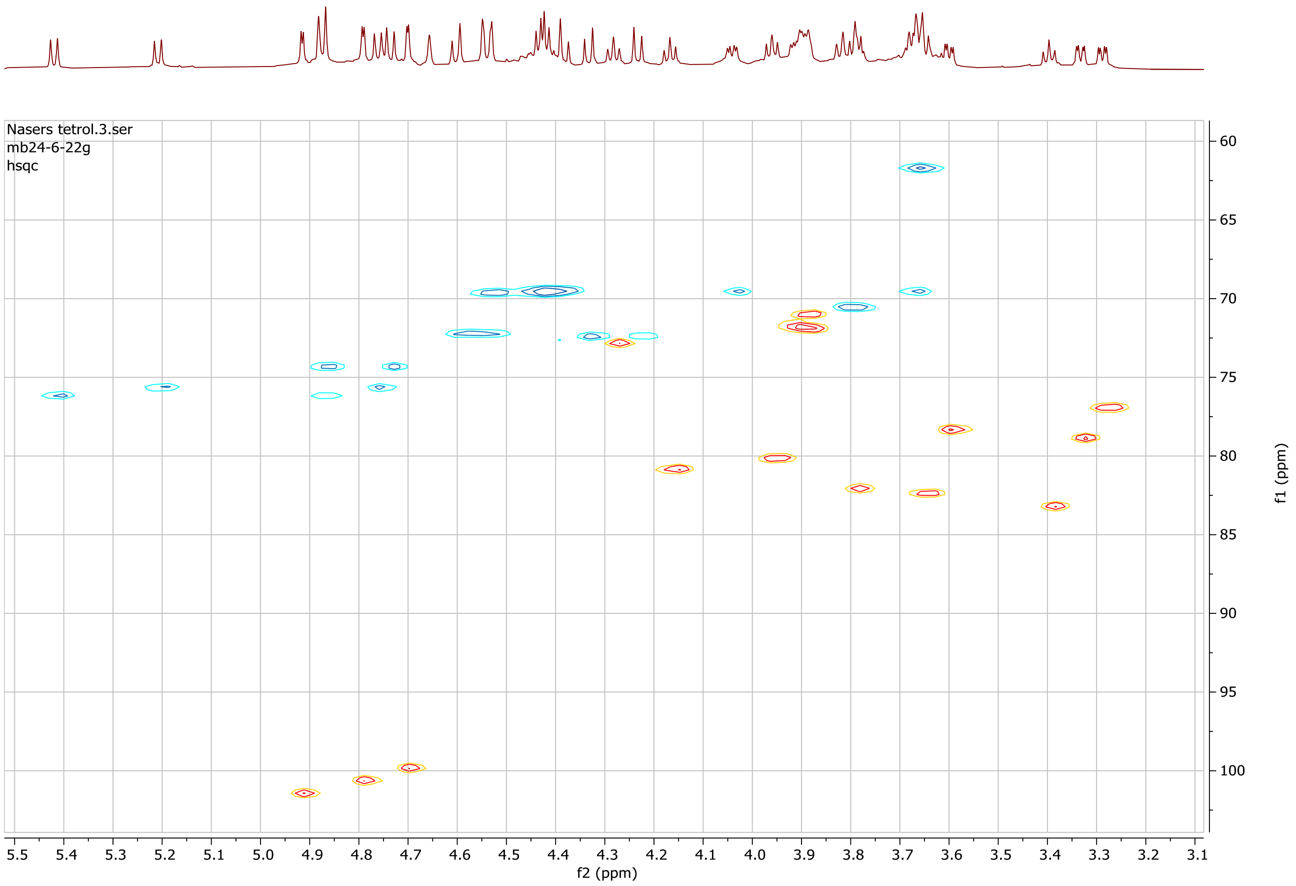

Supplement: File 1 — Copies of NMR spectra of compounds 7–10. [file Beilstein_J_Org_Chem-18-1553-s001.pdf]
